# The Unsafe Inference

**Published:** 1916-09

**Authors:** 


### The Unsafe Inference.

We once proudly observed to a physician who was driving us to a case in a small city: "You must have a considerable country practice"—our pride being in the Sherlockian observation that the thills of his cutter centered on the left edge of the sleigh. The inference was quite wrong. A considerable mass of inferential methods could be gathered together for medicine, all supported by good authority and resting on the most approved principles of inductive reasoning. Detective work is all very well in its way, even as applied to medical practice but, except in stories, inductive reasoning leads us on a great many false clues and the right one is finally found, not so much by continuing to draw inferences, as by accident or by discovery of a definite fact.

Leucocytosis normally rises to correspond to digestive and absorptive activity but it is much more certain and much simpler to investigate digestion itself. It is logical to assume a secondary acid fermentation in urine, to account for certain delayed crystalline deposits, as of uric acid and oxalates but, in a considerable series of tests, we have never been able to make out any rise in acidity on standing, beyond the limit of error of indicators. The theory of an alkaline and an acid tide of urine, converse to acid and alkaline stages of digestion has been taught for half a century but actual titration shows nothing of the sort—which is very natural when we pause to recollect that before the acid stage of digestion is well under way, somewhere near half of the gastric contents have already entered the intestine and started on the alkaline stage. The "highly acid urine of rheumatism" is an expression so trite as to be accepted almost as a matter of course, but actual titration often reveals exceedingly high degrees of acidity in non-rheumatic states and very moderate degrees in acute rheumatism.

There is a prevalent impression that peptic ulcer is due to the burning of a hole in the stomach by an excess of acid, although it is only fair to say that the men who have perhaps given rise to this impression would be the first to ridicule it in this crude form of expression. But on the one hand, a large series of cases of hyperchlorhydria, followed for years, only occasionally yields one of ulcer while, on the other hand, the actual number of tests of stomach contents made at the height of an ulcer is obviously small when we consider the hesitancy to intubate at so critical a period and the statistics of such investigations as have been made, by no means show a regular occurrence of acid excess. Many quasi-scientific and authorative articles on hyperchlorhydria, achylia gastrica, etc., when closely criticised, are found to infer excess of HCl from total acidity or lack of digestive power from very unreliable and imperfect tests.

There used to be a considerable chapter in works on practice on the "sympathetic" relation of the tongue and the stomach. In fact, from inspection of the tongue, all sorts of recondite diagnoses were made. With growing experience, most of this orthodox preface to the practice of medicine, has gradually been dropped from lectures and text books. All sorts of erudition concerning points of pain, dermal states, and various other superficial symptoms have been accumulated—and discarded by the medical men from Missouri.

Laws regarding the heredity of syphilis have been wiped off our statute books since the discovery of the spirochete. The symptomatic classification of malaria has been similarly modified by the direct study of the forms of parasites.

Surgeons used to have a plausible dictum that contraction of the cardia or lower oesophagus, went with a small stomach, contraction of the pylorus with a large organ—perfectly logical and often true, but by no means invariably so.

On different occasions, we have heard equally logical demonstrations of the exclusive association of indicanuria with hyperchlorhydria, and also with hypochlorhydria. And the illogical argument of the burette and test tube is that either condition of the stomach may or may not be associated with indicanuria.

But why multiply examples? Every practitioner must occasionally have found urines of specific gravity too low for sugar, according to the best authority, but nevertheless saccharine.

Any one of these illustrations points the moral for all: Don't infer one condition from something else that ought, logically, to produce a chain of events leading to it. Examine directly for the thing that you want to find out.

